# Shuffle randomization

**DOI:** 10.3325/cmj.2015.56.383

**Published:** 2015-08

**Authors:** Farrokh Habibzadeh, Parham Habibzadeh

**Affiliations:** 1 Shiraz University of Medical Sciences, Shiraz, Iran; 2 Medical Education and Research Center, Petroleum Industry Health Organization, Shiraz, Iran *Farrokh.Habibzadeh@theijoem.com*; 3 Student Research Committee, Shiraz University of Medical Sciences, Shiraz, Iran

In a randomized clinical trial, study participants are assigned to various treatment arms through randomization. This random allocation of participants to treatment arms decreases bias and controls for numerous confounders (1). A common type of randomization is simple randomization, where each participant is randomly assigned to a treatment arm with a known (usually equal) probability, regardless of the treatment assignments of other study participants. For a two-arm trial, that would be achieved by tossing a fair coin. Although in the long run, on average this process will result in approximately equal numbers in each treatment arm, there is no guarantee of equally sized study groups, particularly for small samples. For example, if we want to randomize 10 participants to two treatment arms by flipping a fair coin, there is more than 6% chance of coming up with a severe imbalance as extreme as assigning eight participants to one arm an d two to another arm or worse. Other randomization methods have been proposed to eliminate or at least reduce such extreme imbalance.

Permuted block randomization guarantees that the difference between the two study arms will never exceed half of the block size used (1). We usually use blocks of size four (eg, 1122-1212-2121, etc, where ‘1’ and ‘2’ represent the first or second treatment arm, respectively). Therefore, in the above-mentioned example of 10 participants, using a block size of four, in its worst case, block randomization would result in allocation of six participants to one arm and four to the other. Nonetheless, one of the problems with block randomization is that at certain positions of randomization, say after we randomize three participants to treatment arms, knowing the block size, we can easily figure out the group the fourth participant will be allocated to. Even in certain situations, when the first two participants have been allocated to a given treatment arm, say group 1, we can be sure that the next two participants wil l be allocated to the other arm.

Herein, we propose a new randomization technique that guarantees exact allocation of a pre-specified number of participants to each treatment arm. Suppose we want to randomize 10 participants equally to treatment groups 1 and 2. We begin with a 10-element array of five 1’s and five 2’s as follows: {1, 1, 1, 1, 1, 2, 2, 2, 2, 2}. The value assigned to each element implies the treatment arm to which that element is allocated. Then we shuffle the array elements. Box 1 presents an *R* program function to shuffle an array. Using the algorithm, all elements in the array are swapped with another element at random. In this way, each array element has equal chance of being allocated to each position; hence, random allocation of each array element to the treatment arms.

Box 1 ***R *code for shuffle function**

## This function shuffles the elements of array *x*.

## If the second argument, *seed* is set to a number, the value is used for seeding

## the random generator. Otherwise, a random *seed* is generated based on the system time.

shuffle<-function(x, seed = 0)

{

n<- length(x)

if (seed == 0)

{

seed<- as.integer(Sys.time())

}

set.seed(seed)

}

for (i in 1:n)

{

j<- round(runif (1,1, n))

temp<- x[i]

x[i]<- x[j]

x[j]<- temp

}

return(x)

}

Shuffle randomization is simple and guarantees allocation of exact number of participants to each treatment arm. Using the algorithm mentioned, we can easily decide to randomize participants in another proportion, say seven participants to group 1 and three to group 2 (we need only to shuffle the array {1, 1, 1, 1, 1, 1, 1, 2, 2, 2}). We may also decide to randomize the participants to three groups, say five to group 1, three to group 2, and two to group 3. In the latter scenario we need to shuffle the array {1, 1, 1, 1, 1, 2, 2, 2, 3, 3}.

Shuffle randomization can be considered a specific form of block randomization, if we assume a block size equal to the study sample size. However, unlike block randomization, we can hardly predict allocation of other participants. Alternatively, it can be considered a form of simple randomization as the probability of allocation of participants can precisely be predefined, but it lacks the drawback of imbalance observed with simple randomization.

Running *shuffle* function once on array {1, 1, 1, 1, 1, 2, 2, 2, 2, 2} would result in the array {2, 1, 2, 2, 1, 1, 1, 1, 2, 2}. To test randomness of the array elements, we used *runs.test* function of package ‘*randtests*’ using *R* ver 3.1.2 (2). While the former array (before shuffling) does not have a random distribution (*P* = 0.007), the latter one (after shuffling) does (*P* = 0.502).
